# Experimental Research on Surface Quality of Titanium Rod Turned by Wire Electrical Discharge Turning Process

**DOI:** 10.3390/ma16114009

**Published:** 2023-05-26

**Authors:** Sujeet Kumar Chaubey, Kapil Gupta

**Affiliations:** Department of Mechanical and Industrial Engineering Technology, University of Johannesburg, Doornfontein Campus, Johannesburg 2028, South Africa; schaubey@uj.ac.za

**Keywords:** surface roughness, titanium, wire electrical discharge turning, optimization, wear, SEM

## Abstract

This paper reports the surface quality of a miniature cylindrical titanium rod/bar (MCTB) turned by the wire electrical discharge turning (WEDT) process using a zinc-coated wire of 250 µm diameter. The surface quality was mainly evaluated by considering the very important surface roughness parameters, i.e., the mean roughness depth. A Box–Behnken design (BBD) of the response surface methodology (RSM) based on 17 experimental runs was conducted, where the spark duration “*T_on_*” was found as the most influential parameter affecting the mean roughness depth “*R_Z_*” of the miniature titanium bar. Further, using the grey relational analysis (GRA) technique of optimization, we obtained the least value of “*R_Z_*” 7.42 µm after machining a miniature cylindrical titanium bar with the optimum combination of WEDT’s variable parameters: *T_on_*—0.9 µs, *S_V_*—30 V, and DOC—0.35 mm. This optimization led to a 37% reduction in the surface roughness *R_z_* of the MCTB. The tribological characteristics of this MCTB were also found favorable after conducting a wear test. After completing a comparative study, we can claim that our results are better than those of the past research conducted in this area. The findings of this study are beneficial for the micro-turning of cylindrical bars made from a variety of difficult-to-machine materials.

## 1. Introduction

In recent years, titanium and its alloys, due to being lightweight and strong, have become popular for manufacturing several industrial parts and components [1,2]. Their high specific strength, low elasticity modulus, low density, good weldability, and corrosion-resistant nature have increased their usage in the aerospace, automotive, healthcare, and marine industries. They are also commonly employed in various biomedical applications such as medical implants, tools, and the instruments used in surgery, prosthetics, etc. [3,4]. Some specific parts made from titanium and its alloys include jet engine turbine blades, titanium pipe for the oil industry, computer hard drives and PCB for electronic devices, artificial bones, hip joints endoprosthesis (head, cup, and stem), knee implants endoprosthesis, screws for dental implants, vascular stents, bone fixation (screws and plates), and golf sticks [5,6]. These alloys are considered difficult to machine due to their low thermal conductivity, low modulus of elasticity, high chemical reactivity, high tensile strength, and high hot hardness. Conventional machining of titanium and its alloys is challenging because the common problems encountered are excessive tool wear, high energy and resource consumption, development of thermal stresses and machining force, and poor surface quality, with burrs, tool marks, and cracks on the machined surface [7,8].

The rapidly increasing demand in medical, biomedical, and dental applications for complex cylindrical parts and components equipped with a high aspect ratio and precision motivates researchers to explore and develop alternate machining processes. Turning titanium cylindrical bars, especially of miniature size, is quite difficult by conventional processes due to the requirements of special fixtures and cutting tools [9,10,11]. Wire electrical discharge turning (WEDT) was developed to overcome that difficulty. WEDT is similar to wire electrical discharge machining (WEDM), except for the addition of a rotary axis to hold and rotate the cylindrical bar at a constant speed in clockwise and anti-clockwise directions, for performing straight turning, taper turning, thread cutting, grooving, and other spatial surfaces on difficult-to-machine materials [6]. A controller can control the spindle rotational speed (SRS) of a cylindrical workpiece. Miniature bars, pins, tools, electrodes, and miniature probes are typical cylindrical rotating parts and components that can be manufactured by the WEDT process [12,13].

Some past attempts were made for the WEDT of cylindrical and rotating parts and components from a wide variety of materials. Most of these are based on Inconel and steel cylindrical bars using the belt-driven rotary attachment. The previous research on the micro-turning of miniature titanium cylindrical bars (MCTBs) by WEDT is very limited. Some of the most important research is discussed here. Nag et al. [14] performed the straight turning of an 8 mm diameter cylindrical titanium bar by the WEDT process using a zinc-coated brass wire of 0.25 mm diameter. They concluded that the surface roughness (SR) and wire wear rate decrease with an increase in the spark-off duration, gap voltage, and rotational speed. They claimed to achieve a 1.131 µm average roughness with the optimal parameters. Vignesh and Ramanujam [15] investigated WEDT to perform the straight turning of a 10 mm diameter titanium alloy (grade5) bar using a brass wire of a 0.25 mm diameter. They concluded that the SR decreases with increasing wire speed and spindle rotation speed. Zakaria et al. [16] studied the effect of the WEDT parameters, namely, pulse intensity, gap voltage, wire tension, and SRS on surface roughness for the straight turning of a 9.49 mm diameter titanium alloy bar using a 0.25 mm diameter brass wire. They found that there was deterioration in the surface quality with a decrease in the pulse intensity and gap voltage. Roy and Mandal [17] performed the taper turning of cylindrical bar in multiple stages on a wire spark erosion turning. They found that higher productivity and a better surface finish (i.e., lower values of “R_a_”) can be achieved with higher values of the SRS and wire inclination angle. Naik et al. [18] studied the influence of variable parameters, namely, the spindle rotational speed, spark duration, spark-off duration, servo voltage, wire feed rate, and dielectric flow rate on surface roughness during the turning of an Inconel 718 cylindrical bar by the WEDT process. They found that the surface roughness decreased with an increase in the spark-off duration, servo voltage, and wire feed rate. Cakıroglu and Günay [19] conducted turning on an ASIS L2 tool steel bar by the EDT process using a fabricated copper electrode. They found a 3A discharge current, 8µs spark duration, and 6 µs spark-off duration as the optimum turning parameters, by a GRA analysis, to simultaneously maximize the material removal rate (MRR) and minimize the surface roughness (R_a_) and tool wear rate (TWR). The effect of the electrical discharge machining parameters on the tool wear rate, material removal rate, and surface roughness was analyzed using an analysis of variance [20]. A surface roughness of 3.29 µm was obtained at a machining combination of a 120 μs spark duration, an 8 A current, and a 40 μs spark-off duration. A few more important past attempts highlight the capabilities of electric discharge machining and its variants for machining stainless steel and titanium-type difficult-to-machine materials [21,22].

It can be summarized that the WEDT process, which is an important variant of electric discharge machining, has potential and is required to be explored further to obtain more insights related to the mechanism of turning cylindrical workpieces and securing improved part quality. The following research gaps can be concluded.

- Past work revealed that a belt-driven rotary arrangement was used for the turning of cylindrical workpieces by WEDT. An accurate drive mechanism is still required to be developed for better quality cylindrical part turning by WEDT.
- The average surface roughness *R_a_* of the turned cylindrical surfaces was investigated in most of the past research studies. A gap exists for exploring other more important roughness parameters such as mean roughness depth *R_z_*, which can provide a better indication of the machined surface.
- The more detailed trigological characteristics of the WEDT-machined surface require further investigation.

Considering the identified and aforementioned research gaps, the present research work comprehensively reports the development of a rotary-drive-based WEDT setup and investigation into the turning of miniature bars of titanium (grade5). The major aim of this study is to explore the capability of the WEDT process based on a rotary drive to manufacture precise miniature cylindrical and rotary parts. The specific objectives include to study the effects of the WEDT parameters such as the spark duration, servo voltage, and depth of cut on the mean roughness depth of a Ti grade 2 bar; to find out the most influential parameter among the WEDT parameters; to optimize the WEDT parameters to obtain the least value for the mean roughness depth; and to make a comparison with the past work to analyze the performance of the developed rotary setup for the workpiece rotation by WEDT.

## 2. Experimentation and Measurement

### 2.1. Materials and Machining

Titanium grade5, also known as Ti6Al4V, due to its light weight, excellent strength, heat-treatability, high strength-to-weight ratio, and good corrosion resistance, is extensively utilized in various industrial, domestic, scientific, and commercial applications including in the biomedical field. A cylindrical bar less than 10 mm in diameter is commonly referred to as a miniature or meso-sized bar. In this work, a 60 mm long cylindrical bar of 6 mm diameter made of titanium (grade5) was used as the work material to perform micro-straight turning by the WEDT process. The details of the cylindrical bar made of titanium grade5, along with its chemical composition (by weight) and the levels and values of the variable and the constant parameters of WDET used in the present work, are given in Table 1. WEDT parameters (both variable and constant), their values, and their levels were chosen based on preliminary experiments, suggestions in the literature, and machine constraints. The preliminary experiments included straight turning on a 15 mm long length and at 0.3 mm depth of cut by WEDT on a cylindrical titanium bar of 15 mm diameter, while taking WEDT machine constraints into account. In that preliminary stage, a total of nine experiments were conducted by selecting turning time and wire breakage as the criteria for evaluating WEDT performance. The following conclusions were drawn from preliminary experiments.

- Wire breakage was observed at higher spark duration (more than 1.3 µs) and lower values of spark-off duration (less than 48.5 µs), servo voltage, wire speed (i.e., less than 3 m/min), and dielectric pressure (at 7 Kg/cm^2^).
- Wire vibration took place at the higher values of wire speed due to the long distance between the lower and upper guides.
- Frequent wire breakage took place at the lower value of dielectric pressure (i.e., 7 kg/cm^2^) due to improper flushing of debris from the turning zone (i.e., inter-electrode gap).
- Inaccurate turning (i.e., spiral turning) was observed at lower values of spindle rotational speed (SRS).
- Long turning time was observed at higher values of servo voltage and lower values of spark duration and SRS.

**Figure 1:**
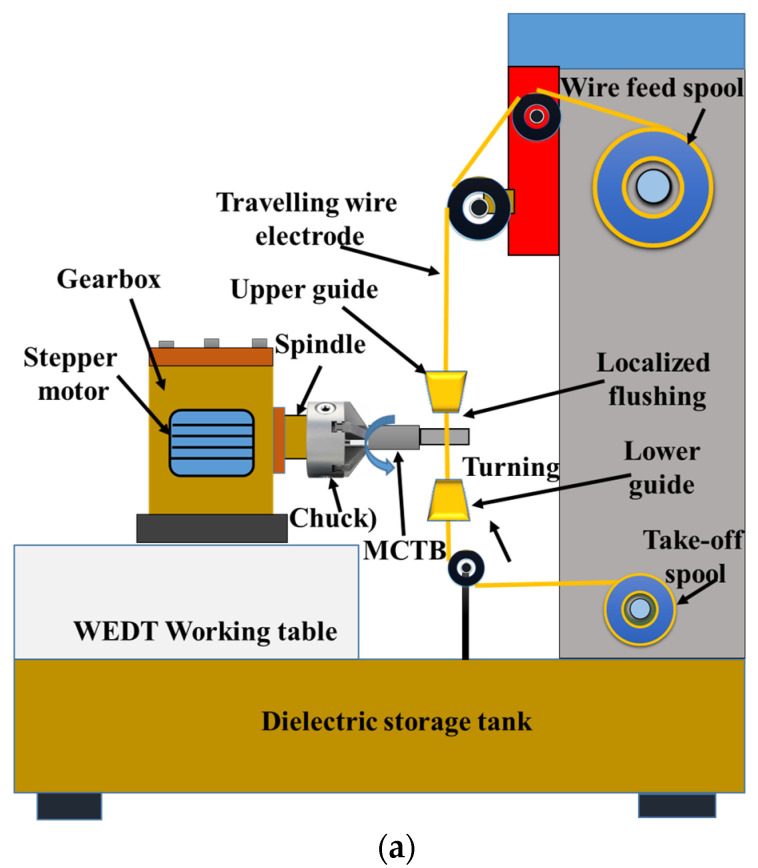

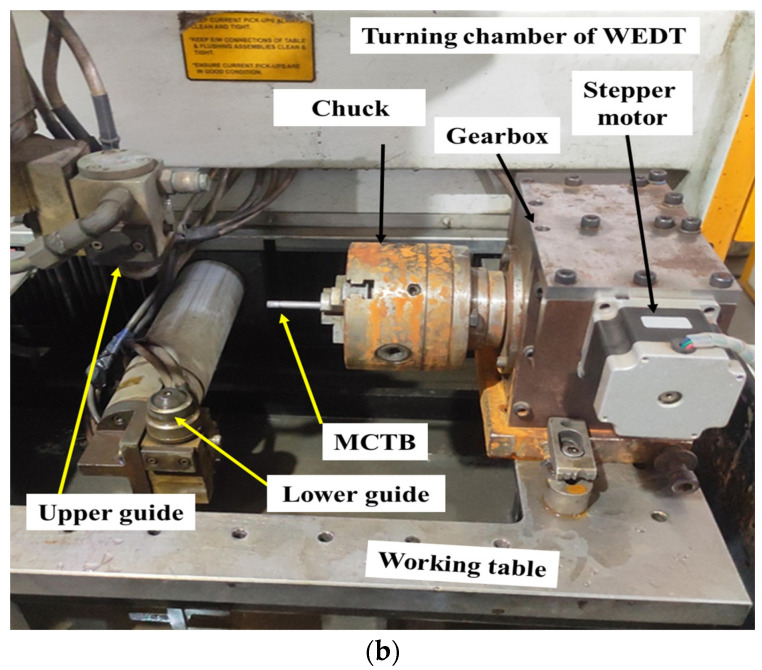
Straight micro-turning of miniature titanium cylindrical bar (MCTB) by WEDT process: (**a**) schematic representation of WEDT and (**b**) enlarged view of turning chamber during micro-turning of MCTB.

A non-submerged-type vertically moving wire-EDM machine tool (model: Sprintcut win; brand: Electronica; country of origin: India) was used to conduct micro-straight turning of miniature titanium cylindrical bar by employing an additional rotating attachment along with a three-jaw chuck, as illustrated in Figure 1. This customized wire-EDM machine is commonly known as wire electrical discharge turning (WEDT). The purpose of rotary attachments, which contain a three-jaw chuck, a gearbox, and a stepper motor, is to securely hold the cylindrical bar and rotate it at a certain rotational speed to carry out the turning operations by the wire electric discharge machining.

The stepper motor is connected to the control panel, which comprises a power supply unit, a power drive unit, and a speed controller. The spindle speed of a rotary attachment can be adjusted by using a DC motor controller. A very fine, soft, zinc-coated brass wire of 250 µm diameter and 800 N/mm^2^ tensile strength was employed as a tool electrode for turning miniature cylindrical titanium bar (MCTB) by WEDT in the presence of deionized water, which serves as a dielectric medium. This customized computer numerically controlled (CNC) 4-axis (X, Y, U, and V) machine can achieve wire inclination angle up to 30^0^. Wire tension/rigidity is only maintained between the upper and lower guides to avoid wire vibrations.

### 2.2. Experimental Procedure and Measurement

The response surface methodology’s Box–Behnken design (BBD) was used to prearrange and carry out experimental runs accordingly. BBD is a rotatable design that only needs three levels for each parameter being taken into consideration. Spark duration *T_on_*”, Servo voltage “*S_V_*”, and depth of cut “*DOC*” are the three variable process parameters considered in this work. Based upon the variation of these 3 parameters at 3 levels each, 17 experimental combinations were designed according to BBD to conduct micro-turning operations on cylindrical titanium bar (MCTB), and each experimental run was replicated twice. As a result, a total of 34 micro-turning experiments were conducted by WEDT to perform step micro-turning on miniature cylindrical titanium bars (MCTBs). Replicating each experiment had the goal of reducing the influence of uncontrolled variations such as experimental error and enhancing the statistical precision of the experimental runs. The results of these experimental runs were used to evaluate the influence of three selected varying WEDT parameters through graphical representation.

Surface roughness is one of the key indicators in determining the quality of any surface, by knowing the amount of irregularity [23]. It indicates the possible behavior of any surface when it comes in contact with another. Wear characteristics and tribological behavior are very much dependent on surface quality. One of the most important roughness parameters is the mean roughness depth “*R_Z_*”, which was considered as the output response to evaluate the surface quality of MCTBs machined by WEDT in this work and determine the potential of WEDT for the micro-turning of MCTB. The mean roughness depth is superior to average roughness and maximum roughness, in the sense that it gives a real indication of the surface irregularity, since it is the average of the maximum roughness (distance between the highest peak and deepest valley) values for all five equal segments of the evaluation length. Mean roughness depth “*R_Z_*” is the output parameter selected to evaluate the surface quality of the cylindrical bar. The turned surface of the cylindrical titanium bar with a minimum value of mean roughness depth “*Rz*” is considered the best surface quality of the turned MCTB in this study.

**Figure 2:**
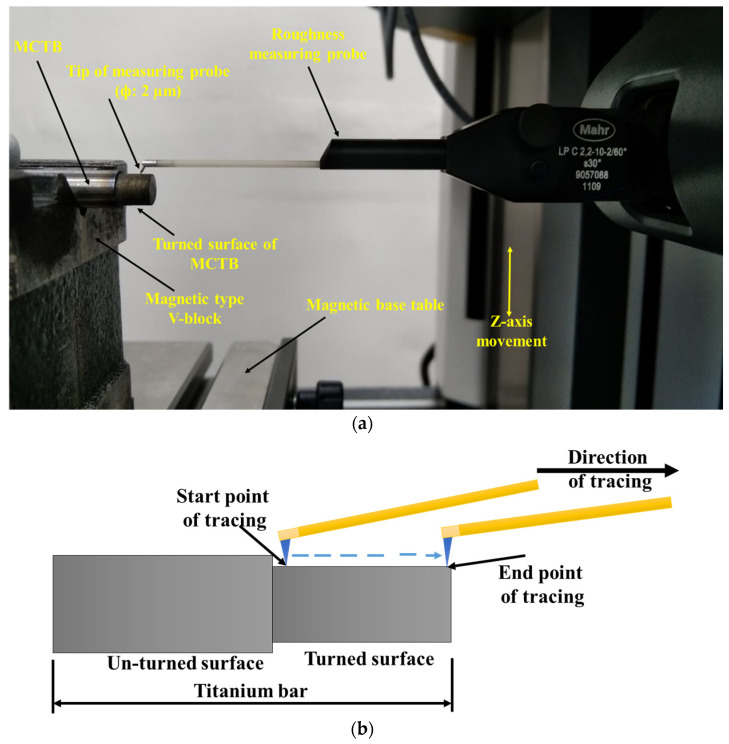
Roughness measurement of turned MCTB by LD 130 roughness tester: (**a**) LD 130 roughness tester and (**b**) procedure of roughness measurement.

Figure 2 illustrates the step-by-step procedure of roughness measurement of turned MCTB by LD 130 roughness tester. Mahr metrology (Germany) made LD-130 3D roughness cum contour tester that was used to measure the surface roughness of turned surfaces of MCTBs with help of a 2 µm diameter probe. Roughness measurements were conducted following the ISO 4287 and using a 0.8 mm cut-off length, 2 mm evaluation length, and Gaussian filter. Three roughness measurements were taken at different points along the turning length for each MCTB that was turned at different parametric combinations of WEDT, according to the designed experimental combinations in each run, and their average values (i.e., Trial1 and Trial2) were considered.

Coefficient of friction (COF) can reveal the status of surface irregularity to a great extent, and its value for rough surface is always high [23]. It is one of the important parameters that is considered to evaluate the wear behavior and tribological characteristics of the surface under examination.

To test this, a linear reciprocating wear testing machine (model: CM9104 from Ducom, USA) was used to examine the tribological fitness of the turned MCTBs. Wear tests were conducted under dry conditions at room temperature by sliding a 6 mm diameter tungsten carbide ball (950 HV 9 hardness) for a 5 mm distance at 15 Hz frequency for 15 min time duration under 15 N normal load on a WEDT-turned surface of MCTB sample. A field-emission scanning electron microscope (FE-SEM) SUPRA 55 from Carl Zeiss (Germany) was also used for MCTB surface quality evaluation. 

## 3. Results and Discussion

Combinations of the selected WEDT variable parameters for all 17 experimental runs and their corresponding values (i.e., Trial1) of mean roughness depth “*R_Z_*” are tabulated in Table 2 along with their replication (i.e., Trial2) values. The average values (i.e., the average of Trial1 + Trial2) of *R_Z_* were used for an analysis using statistical software, Design Expert trial version.

### 3.1. Effect of Parameters on Responses

**Figure 3:**
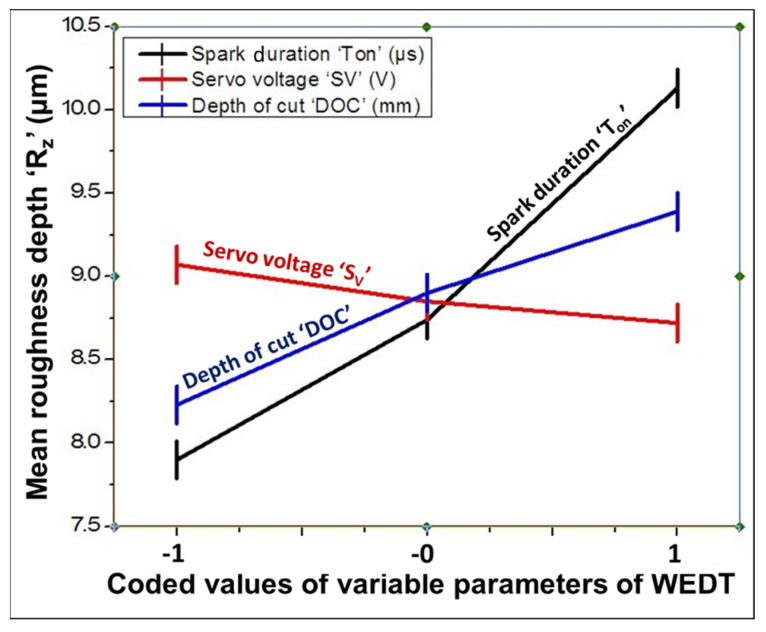
Influence of selected variable parameters on mean roughness depth of miniature cylindrical titanium bar after micro-turning by WEDT.

The influence of the considered WEDT variable parameters, namely, spark duration “*T_on_*”, servo voltage “*S_V_*”, and depth of cut “DOC” on mean roughness depth “*R_Z_*” is discussed with the help of a graphical representation, as shown in Figure 3. In these graphs, the abscissa depicts the values of the mean roughness depth in µm, whereas the ordinate shows the values of the WEDT variable parameters. The following observations are drawn from Figure 3.

- The mean roughness depth is continuously increasing with an increase in the spark duration.
- The mean roughness depth is continuously decreasing with an increase in the servo voltage. However, higher values of the servo voltage also increase the turning time.
- The mean roughness depth continuously increases with the increase in the depth of cut.
- A better surface finish (i.e., minimum values of *R_Z_*) occurs at parametric combination 0.9 µs T_on_ and 30 V S_V_ (i.e., experimental run 10), as shown in Table 2 and Figure 3.

The spark duration is the period of spark occurrence in microseconds between the workpiece being machined (cylindrical Ti grade 5 bar in the present case) and the vertically traveling zinc-coated wire [24,25]. Therefore, higher values for the spark duration (1.3 µs) generate sparks for longer periods. A longer duration of the spark time corresponds to the availability of the discharge energy for a longer period of time and is responsible for the formation of the non-uniform deeper and wider craters on turned surfaces, resulting in a deterioration in the work surface quality in terms of a poor surface finish and, hence, the higher values for the mean roughness depth.

The servo voltage determines the inter-electrode gap (IEG), which must be maintained during the machining process between the wire and the workpiece to avoid short-circuiting, and the wire breakage and ensures the proper flushing of debris from the turning zone [26,27]. The IEG significantly affects the concentration of sparks in the turning zone during micro-turning by WEDT. Thus, a larger IEG can be maintained at a higher level of servo voltage. Therefore, *R_Z_* decreases with an increase in the IEG at higher levels of servo voltage due to the formation of quite stable sparks and the proper flushing of debris from the turning zone. Inconsistent turning due to the poor utilization of sparks in the turning zone takes place for the IEG values obtained at voltage levels over 30 V. Furthermore, a very low value (i.e., less than 20 V) for the servo voltage leads to short-circuiting, wire breakage, and the improper flushing of debris from the turning zone due to the corresponding low values of the IEG.

The mean roughness depth increases with an increase in the depth of cut for its given range in this study. The distance traveled by the wire along the cross-section of the cylindrical (i.e., along its diameter) bar is known as the depth of cut, whereas the distance traveled by the wire along the axis of the cylindrical bar is known as the turning length. Both the depth of cut and turning length are expressed in mm. The depth of cut indicates the total amount of diameter reduction per pass of the wire during the straight micro-turning of the MCTB by WEDT. Specifically, it is half of the difference between the cylindrical bar’s diameters and is mathematically expressed by equation 1. The mean roughness depth increases with an increase in the depth of cut from 0.2 to 0.5 mm. At a higher depth of cut, a longer length of the wire comes in contact with the cylindrical bar, which affects the removal of debris from the inter-electrodes gap (i.e., the turning zone). Due to this, the deposition of removing particles takes place on the turned surface and, thus, affects the surface quality of the turned surface and smooth turning due to a reduction in the standard inter-electrodes gap and a longer distance between the upper and lower guides during turning.

(1) $$Depth\;of\;cut\;\left(DOC\right)=\frac{Bar\;diameter\;before\;turning-Bar\;diameter\;\;after\;turning}{2}$$

The analysis of variance (ANOVA) results for the effect of the variable parameters of WEDT on the mean roughness depth revealed that (i) the developed prediction model for mean roughness depth “*Rz*” is significant and was identified by using a 95% confidence interval of *p*-values (i.e., *p*-values < 0.05); (ii) spark duration is the most significant variable parameter of WEDT for the micro-turning of the MCTB; and (iii) the lack of fit test is non-significant. Therefore, this indicates that the developed prediction model for *Rz* accurately fits the experimental data.

**Figure 4:**
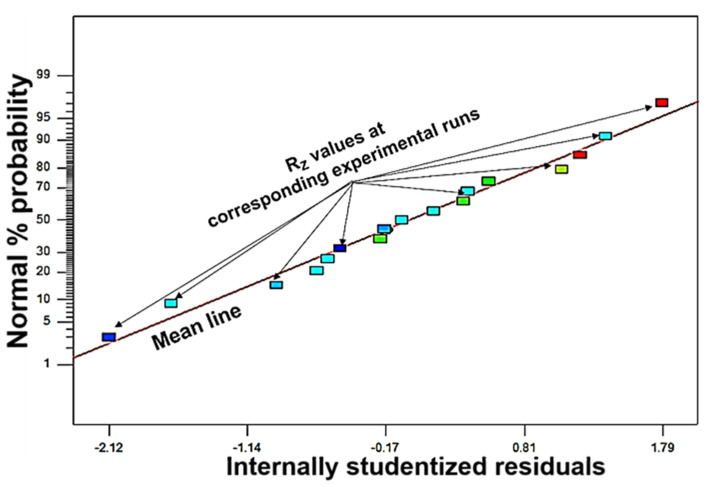
Normal percentage probability distribution graphs of residuals for mean roughness depth of miniature cylindrical titanium bar after micro-turning by WEDT.

The values of the mean roughness depth or the residuals for all the experimental runs accumulated around the mean line, i.e., the straight red line of the normal percentage probability graph, as shown in Figure 4. This mean line is used for the analysis of the residuals. It reveals the minimum error in the experimental results, as the residuals are accumulated around the mean line. Therefore, this graph confirms that the residuals of all the experimental runs are normally distributed.

### 3.2. Optimization

The optimization of machining parameters is essential to obtain the ideal results with higher productivity in machining processes. A specific machining process can produce better machining results with equal resources by optimizing the machining parameters [28]. In this study, a grey relational analysis (GRA) was used to find out the optimum turning combination of WEDT for the mean roughness depth from the experimental run data, as shown in Table 2. In the GRA, the experimental values of the mean roughness depth “*R_Z_*” and “*Y_ij_*” (where “” is the index for “n” number of responses, i.e., *i =* 1, 2,…., *n*; and “*j*” is the index for “m” number of experiments, i.e., *j =* 1, 2*,…., m*);) are normalized as “*Z_ij_*” (where “*Z_ij_*” is the normalized value of “*Y_ij_*”) within a specified range of 0 to 1. Per the GRA, Equation (2) is utilized to minimize (the lower the better) the mean roughness depth [29,30].

Minimizing the mean roughness depth “R_Z_” (the lower the better)

(2) $$Z_{ij}=\frac{max\left(Y_{i}\right)-Y_{ij}}{max\left(Y_{i}\right)-min\left(Y_{i}\right)}$$

where *max (Y_i_)* and *min (Y_i_)* are the highest and lowest values of the *ith* response among the 17 experimental runs, as shown in Table 2. The grey coefficient (GC), based on normalized data, shows a correlation between the desired and actual values of the response. Using normalized data, Equation (3) is utilized to calculate the grey coefficient “*GC_ij_*” for the *ith* response of the *jth* experiment [29].

(3) $$GC_{ij}=\frac{\;\Delta_{min}+\alpha \;\Delta_{max}\;}{\Delta_{ij}+\;\alpha \;\Delta_{max}}$$

where “α” refers to the distinguishing coefficient, which has a 0 to 1 range of values. Since there is only one response considered, i.e., the mean roughness depth, in this work, therefore, the assigned value of α is 1. “Δ*_ij_*” indicates the inclination of the i*th* response of the *jth* experimental run from its anticipated value. It is equivalent to the absolute variation between the ideal normalized value “*Z_ii_*” and the normalized value “*Z_ij_*” of the *ith* response, i.e., $\Delta_{ij}=\left|Z_{ii}-Z_{ij}\right|$, while Δ_min_ and Δ_max_ are the minimum and maximum values of Δ_ij_. The GRG of the *jth* experiment “*G_j_*” is computed by considering the average values of the grey coefficient “*GC_ij_*” of all the responses corresponding to the *jth* experimental run in Equation (4).

(4) $$G_{j}=\frac{1}{n}\;\overset{n}{\underset{i=1}{\sum }}GC_{ij}$$

where “*n*” is the total number of responses. The maximum value of “*Gj*” indicates the optimum parametric combination of WEDT for the micro-turning of the MCTB. In this study, experimental run 10 has a maximum value of “*Gj*”. Thus, the *parametric* combination of experimental run 10 is identified as the optimum parametric combination (i.e., 0.9 µs as the *T_on_*, 30 V as the *S_V_*, and 0.35 mm as the *DOC*) of WEDT, and 7.15 µm is identified as the best value of the mean roughness depth. However, it requires further validation. Thus, two confirmation experiments were carried out at 0.9 µs *T_on_*, 30 V *S_V_*, and 0.35 mm *DOC* to verify the results of the GRA optimization. The optimal turning combination for the CERs is listed in experimental run 10, which has higher values for the grey relational grade (GRG), as shown in Table 2. The average values of *R_Z_* were used to calculate the difference between the confirmation and the optimum results obtained from the GRA. Table 3 presents the optimum values of the turning combination and the corresponding values of the mean roughness depth obtained during the confirmation experiments.

It can be observed from Table 3 that the average value of the mean roughness depth is 7.42 µm, which is close to the expected value of 7.15 µm with a variation of 3.78%. It can be said that there is a good agreement between the GRA prediction and the results of the confirmation experiments, which indicates the successful optimization of the WEDT process obtains the best value for the mean roughness depth. When compared with the maximum *R_z_* value, i.e., 11.74 µm for experiment number 12, which corresponds to the lowest GRG, the reduction in the surface roughness is approximately 37%.

### 3.3. Surface Morphology Examination

The roughness measurements, wear test, and FE-SEM examination of the miniature titanium bar machined at the optimum parameters of WEDT are discussed in this section. 

#### 3.3.1. Surface Roughness Measurement

**Figure 5:**
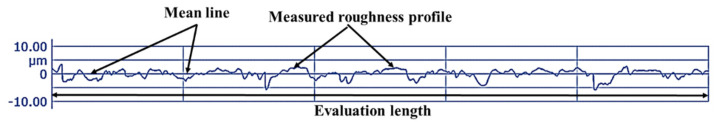
Profile of turned surface of the best finished cylindrical titanium bar turned by WEDT at optimum condition.

Three roughness measurements were performed at different points on the turned cylindrical surface along the turning length for each confirmation run, and the mean values of the mean roughness depth (i.e., Trial1 and Trial2) were taken into account. The measurement results revealed a mean roughness depth R_Z_—7.42 µm. The average values of skewness and the kurtosis-type tribology parameters are −1.07 and 4.03, respectively. The negative value of skewness indicates that the turned surface of the MCTB has good bearing strength and, hence, good tribological characteristics. Figure 5 illustrates the 2D roughness profile obtained by the roughness measurement of the surface of the MCTB machined at the optimum WEDT parameters.

#### 3.3.2. Wear Testing and Analysis

**Figure 6:**
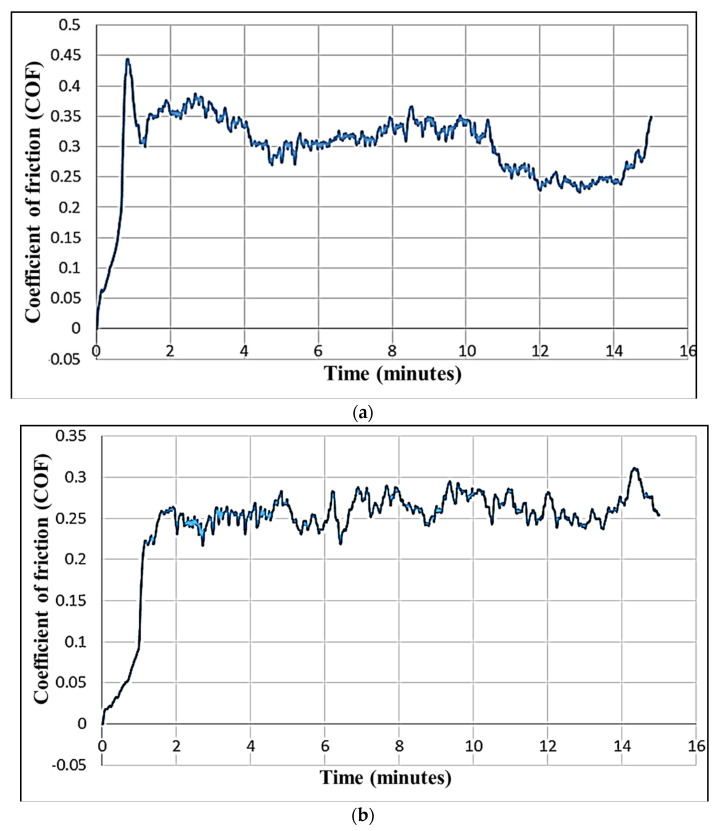
Variation of COF with time during wear testing of miniature cylindrical titanium bar (MCTB) micro-turned by WEDT: (**a**) micro-turned MCTB with highest mean roughness depth and (**b**) MCTB with least mean roughness depth micro-turned at optimal WEDT parameters.

The coefficient of friction (COF), which is a ratio of the friction and normal forces, is affected by the stickiness and roughness of two surfaces. Although a nominal value of the COF is essential, for most applications, it is desirable to keep it low [14]. The COF curves for the poor surface-finished MCTB (i.e., a higher value of the mean roughness depth of the MCTB; experimental run 12) and the optimum MCTB (i.e., a better-turned surface-finished MCTB; experimental run 10) are shown in Figure 6a,b. These curves show how the COF varies with the fretting wear duration. It is evident from Figure 6a that the COF initially increases, goes beyond the value of 0.4, and then decreases to 0.3. Further, there are lots of variations in the COF from 4 to 15 min. At a fretting time of 15 min, the maximum value of the COF is about 0.35. While, for the entire duration of 15 min, the maximum value of wear reaches up to 0.45. The instability and variation in the COF are caused by the non-uniform and rough cylindrical surface of the MCTB turned by WEDT due to wire vibration, improper flushing, and unstable turning. On the other hand, for the MCTB turned at the optimum turning parameters, the COF variation with the wear time ranges at lower levels, and the maximum value reaches up to 0.32 (Figure 6b). The reason behind this is the better surface characteristics of the WEDT-turned MCTB with less irregularity.

#### 3.3.3. Scanning Electron Microscopy

**Figure 7:**
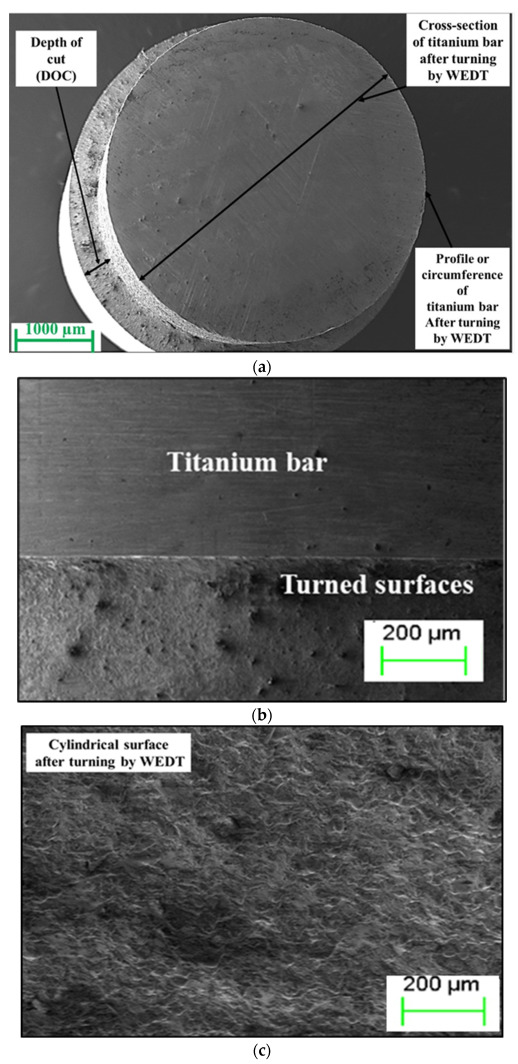

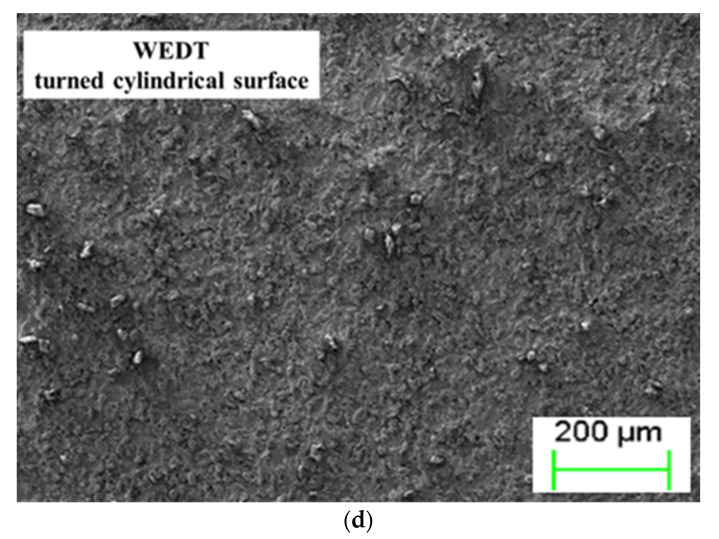
Scanning electron micrographs of (**a**) miniature cylindrical titanium bar, and (**b**–**d**) its surface, after turning by WEDT at the optimal conditions.

High-magnification micrographs were captured using an FE-SEM to investigate the WEDT-turned surface of the MCTBs. The MCTB machined at the optimum WEDT parameters and equipped with the best mean roughness depth was cleaned in acetone before an SEM examination. As evident from Figure 7a–d, the machined surface reveals the formation of shallow and regular craters. As such, no serious damage on the surface due to cracks, globules, and voids is observed. The cross-sectional geometrical profile of the MCTB and its corner edge are found to have no serious flaws/errors.

### 3.4. Comparison with the Past Studies

The research findings from this study are compared with the past work completed on WEDT, and the important points are as follows.

- In the past, no work was reported on the wear analysis of the WEDT-turned surface of a cylindrical bar. We improved the wear characteristics of a miniature titanium bar after optimization.
- The mean roughness depth *R_z_*, which is the superior parameter of this surface quality investigation and highlights the clear picture of the machined surface, was not considered in the past work on the wire EDM-based turning of cylindrical bars. In previous research, the average roughness “*R_a_*” and the maximum roughness “*R_max_*” were evaluated [14,16]. After optimization, we achieved a 7.42 µm R_z_.
- In this study, we successfully performed the micro-turning operation on a 6 mm diameter cylindrical titanium bar, which is smaller than the cylindrical bars employed in the previous work [14,16,18,31].
- In terms of the effects of the WEDT process’s parameters, the pulse-on time or spark duration was also found to be the most influencing parameter, with higher values that led to surface quality deterioration [14,16].
- The superior results of this study indicate that the gearbox configuration we used in the rotary arrangement to rotate the workpiece is more accurate than the belt-driven rotary attachment configuration utilized in the previous work, because slipping is a big issue in belt- and pulley-driven transmission systems [32,33].

## 4. Conclusions

In this study, we successfully turned a miniature cylindrical grade 5 titanium bar with a 6 mm diameter using WEDT. The following findings from this study can be highlighted.

- The mean roughness depth “*R_Z_*” steadily increases with an increase in the spark duration “*T_on_*” and the depth of cut “*DOC*”. However, it decreases with an increase in the servo voltage “*S_V_*”.
- The spark duration “*T_on_*” is found to be the most influencing variable parameter of WEDT for the mean roughness depth “*R_Z_*”.
- The combination of the WEDT parameters of 0.9 µs *T_o_*_n_, 30 V *S_V_*, and 0.35 mm DOC were found to be the optimum for the micro-turning of the miniature titanium bar.
- The MCTB turned by WEDT in the confirmation experiments has a 7.42 µm as an average value of the mean roughness depth “*R_z_*”, which is much closer to the optimum mean roughness depth of 7.15 µm, as predicted by the GRA with only a 3.78% error.
- The MCTB turned at the optimum parameters was found to be better in tribological performances compared to the MCTB turned under a non-optimal regime.
- An SEM examination of the MCTB turned at the optimum parameters revealed a uniform geometrical profile and a better cylindrical surface with low surface irregularity and, as such, no serious concerns related to wire marks, burrs, or other defects.
- The gear-driven rotary attachment to ensure a constant rotational speed of the cylindrical bar during the worked well and was identified as a superior substitute for the conventional belt-driven WEDT processes.
- This study is helpful for industry users and researchers to explore turning and other operations to develop miniature pins, rods, bars, and screws for a wide range of industrial applications. Most importantly, the selection and setting of the WEDT parameters secure the best surface quality for the machined parts.
- The following points present some important avenues for future research:
- Development of an inbuilt rotary setup with the work table for WEDT;
- Investigations into the sustainability of the wire spark erosion turning process;
- Soft computing-based optimization of WEDT;
- Investigations into steeped turning, taper turning, thread cutting, and grooving by WEDT.

## Figures and Tables

**Table 1:** Details of selected WEDT variable parameters and specifications of the cylindrical bar.

WEDT Variable Parameters				Responses
Name, “Symbol”, and (Unit)	Actual (Coded) Levels			
	Low (−1)	Medium (0)	High (1)	
Spark duration “*T_on_*” (µs)	0.9 (−1)	1.1 (0)	1.3 (1)	Surface roughness parameters: Mean roughness depth “*R_Z_*” In situ responses: Turning time Other: Wear analysis, SEM
Servo voltage “*S_V_*” (V)	20 (−1)	25 (0)	30 (1)	
Depth of cut “*DOC*” (mm)	0.2 (−1)	0.35 (0)	0.5 (1)	
Constant parameters				
WEDT parameters: peak current “*I_P_*”: 12 A; spark-off duration “*S_off_*”: 48.5 µs; wire speed “*W_S_*”: 3 m/min; wire tension “*W_T_*”: 1140 g; flushing pressure “*W_P_*”: 15 kg/cm^2^; cutting speed “*C_S_*”: 100%; spindle rotational speed: 50 rpm Workpiece and wire: workpiece type: cylindrical bar; workpiece material: titanium (grade5); electrode material: zinc-coated wire; wire diameter: 0.25 mm; wire tensile strength (WTS): 800 N/mm^2^ Dielectric: deionized water; dielectric conductivity: 20 mho				
Detailed specifications of the cylindrical bar				
Type: miniature; bar diameter: 6 mm; total length of bar: 60 mm; turning length: 5 mm				
Chemical composition of cylindrical titanium bar				
Ti: 90%; Al: 5.5–6.75%; V: 3.5–4.5%; Fe: <0.4%; C: <0.08				

**Table 2:** WEDT parameter combinations for experimental trials and corresponding values of mean roughness depth with grey relational grade values.

Ex. Runs	WEDT Variable Parameters			Responses			Grey Relational Grade (GRG)
	Spark Duration “*T_on_*” (μs)	Servo Voltage “*S_V_*” (V)	Depth of Cut “DOC” (mm)	Mean Roughness Depth “*R_Z_*” (µm) of the Cylindrical Bar after Turning Avg. (Trial1 + Trial2)			
				Trial1	Trial2	Avg.	
1	0.9 (−1)	20 (−1)	0.35 (0)	09.29	10.26	07.78	1.04
2	1.1 (0)	25 (0)	0.35 (0)	07.86	08.09	09.98	0.67
3	1.1 (0)	30 (1)	0.2 (−1)	12.89	13.01	08.45	0.89
4	0.9 (−1)	25 (0)	0.5 (1)	10.09	10.55	08.32	0.92
5	1.3 (1)	25 (0)	0.2 (−1)	08.26	08.75	07.51	1.12
6	1.1 (0)	25 (0)	0.35 (0)	12.88	13.05	07.91	1.01
7	1.1 (0)	20 (−1)	0.5 (1)	07.82	08.54	08.18	0.95
8	1.1 (0)	25 (0)	0.35 (0)	07.67	08.88	08.28	0.93
9	1.1 (0)	20 (−1)	0.2 (−1)	10.60	10.58	08.59	0.86
**10**	**0.9 (−1)**	**30 (1)**	**0.35 (0)**	10.22	09.87	**07.15**	**1.24**
11	1.1 (0)	25 (0)	0.35 (0)	08.79	07.61	08.20	0.94
**12**	**1.3 (1)**	**20 (−1)**	**0.35 (0)**	10.18	9.29	**11.74**	**0.52**
13	1.3 (1)	25 (0)	0.5 (1)	11.72	11.60	11.66	0.52
14	1.1 (0)	30 (1)	0.5 (1)	09.95	09.33	09.64	0.71
15	1.1 (0)	25 (0)	0.35 (0)	09.22	09.68	09.45	0.73
16	0.9 (−1)	25 (0)	0.2 (−1)	10.96	11.78	08.37	0.91
17	1.3 (1)	30 (1)	0.35 (0)	11.44	11.82	09.63	0.71

**Table 3:** GRA-based optimum parameters of WEDT and results of confirmation experiments.

Variable Parameters of WEDT		Optimized Value by GRA	Experimental Validation (i.e., Close Standard Values Available in WEDT)			Error (%)
			Trial1	Trial2	Avg. Values of Trial1 + Trial2	
WEDT process parameters	*T_on_* (µs)	0.9	0.9			
	*S_V_* (V)	30	30			
	*DOC* (mm)	0.35	0.35			
Responses	*R_Z_ (*µm*)*	7.15	7.22	7.61	7.42	3.78

## Data Availability

Data will be available on request.
